# Female Sex Worker Social Networks and STI/HIV Prevention in South China

**DOI:** 10.1371/journal.pone.0024816

**Published:** 2011-09-13

**Authors:** Joseph D. Tucker, Hua Peng, Kaidi Wang, Helena Chang, Sen-Miao Zhang, Li-Gang Yang, Bin Yang

**Affiliations:** 1 Infectious Disease Unit, Massachusetts General Hospital, Boston, Massachusetts, United States of America; 2 Guangdong Provincial STD Control Center, Guangzhou, China; 3 Harvard Medical School, Boston, Massachusetts, United States of America; 4 Urology Department, University of California San Francisco, San Francisco, California, United States of America; University of Florida, United States of America

## Abstract

**Background:**

Reducing harm associated with selling and purchasing sex is an important public health priority in China, yet there are few examples of sustainable, successful programs to promote sexual health among female sex workers. The limited civil society and scope of nongovernmental organizations circumscribe the local capacity of female sex workers to collectively organize, advocate for their rights, and implement STI/HIV prevention programs. The purpose of this study was to examine social networks among low-income female sex workers in South China to determine their potential for sexual health promotion.

**Methods/Principal Findings:**

Semi-structured interviews with 34 low-income female sex workers and 28 health outreach members were used to examine how social relationships affected condom use and negotiation, STI/HIV testing and health-seeking behaviors, and dealing with violent clients. These data suggested that sex worker's *laoxiang* (hometown social connections) were more powerful than relationships between women selling sex at the same venue in establishing the terms and risk of commercial sex. Female sex workers from the same hometown often migrated to the city with their *laoxiang* and these social connections fulfilled many of the functions of nongovernmental organizations, including collective mobilization, condom promotion, violence mitigation, and promotion of health-seeking behaviors. Outreach members observed that sex workers accompanied by their *laoxiang* were often more willing to accept STI/HIV testing and trust local sexual health services.

**Conclusions/Significance:**

Organizing STI/HIV prevention services around an explicitly defined *laoxiang* social network may provide a strong foundation for sex worker health programs. Further research on dyadic interpersonal relationships between female sex workers, group dynamics and norm establishment, and the social network characteristics are needed.

## Introduction

Unsafe sex is the second most important risk factor for disability and death in low-income areas [1] and unprotected sex within the commercial sex industry is likely a major driver of HIV/STI transmission in these regions [2], [3]. Several studies have identified social influences as important determinants of condom use among female sex workers [4], [5], [6], [7]. Reducing harm associated with sex work requires an understanding of the social and cultural context of commercial sex [8], [9]. Sex worker mobilization through nongovernmental organizations (NGOs) is dependent on the local context, much more common in India, Canada, the United States, and other places with an active civil society [10], [11] and less common in China [12], Singapore, and Vietnam. Here we define civil society as self-governing, non-state associations that identify and address collective interests [13]. Both model public health programs and sex work research have assumed the presence of NGOs to serve vulnerable sex workers since government-affiliated organizations may not be trusted or closely connected to female sex workers. This has generated a paradox whereby most sex workers in low-income areas are not members of an NGO and are not interested in creating new social organizations hinging on their identity as sex workers, yet most HIV/STI programs for sex workers have been developed in the context of NGOs and a vibrant civil society.

Although female sex workers in China are unlikely to have a local NGO to position STI/HIV prevention programs [14], there are social networks that could be leveraged to promote sexual health in China and elsewhere. Rural Chinese women often migrate to urban regions in search of higher paying jobs, but then encounter adversity in urban areas in identifying and maintaining these positions [15]. Common themes in the demographics (e.g., young women) and migration patterns (e.g., rural to urban) of female migrant women in China foster the development of social ties [16]. These “hometown” (*laoxiang*) social ties are important in the Chinese context [17], [18] and may serve to make an otherwise challenging urban environment more reasonable [17]. In the context of our research, these *laoxiang* ties are defined by women coming from the same region who migrate to the same municipality. Social science research has shown how these *laoxiang* social ties provide durable social cohesion among labor protesters [19], [20], entrepreneurs and business leaders [21], women [22], and other groups [22], [23].

The migrant life experience may exacerbate sexual risk for *laoxiang* in the Chinese context. China has an alarming burden of syphilis [24], [25], [26] and increasing sexually transmitted HIV in some regions [27], [28]. A substantial number of female sex workers in China are rural-to-urban migrants [29], [30] and rural-to-urban migration among a subset of female migrants may increase sexual risk in China [31], [32], [33]. The experience of rural-to-urban migration for women in China can distance them from home-based social norms and situate them in an environment that promotes sexual risk taking [32]. Furthermore, female migrant sex workers may have problems accessing health insurance and other local resources away from their official rural residence [17]. China's health insurance system is undergoing reform [34], but the extent to which female migrants have access to basic health services in urban areas is limited [35], [36]. While female rural-urban migration cannot be said to universally increase STI/HIV risk, there is a subset of poor female migrant women who have a higher sexual risk in their new urban environment.

A diverse literature supports that Chinese sex workers heavily rely on structural cues and social support in considering condom use [37], [38], [39], [40], but an effective model STI/HIV prevention program for sex workers in China has eluded behavioral researchers and public health officials to date [41]. Qualitative research may be particularly useful for better understanding behavioral sex worker interventions since decisions about condom use reflect a complex interplay of individual, community, and social factors [42]. There have been no studies in China focused on how social networks could be used to promote sexual health among female sex workers. The goal of this study was to investigate the influence of *laoxiang* social networks on female sex worker condom use, capacity to deal with anti-prostitution campaigns, and health seeking behaviors.

## Methods

This qualitative analysis included both semi-structured interviews of female sex workers and health outreach members, in addition to direct observation at entertainment centers where sex is sold. The interviews were conducted among these groups to help bridge the gap between vulnerable female sex workers and the outreach members who are responsible for STI/HIV prevention. The Institutional Review Boards at the Harvard University Faculty of Arts and Sciences and the Guangdong Provincial Center for STI Control approved this research. All participants provided verbal consent because there was no more than minimal risk associated with participating in this study and no biological samples were collected. All interviewers signed forms indicating that verbal consent had been obtained for each participant. This consent procedure was approved by both IRBs prior to study launch.

Increasingly social and environmental factors are recognized as critical in establishing a risk environment in which female sex workers live and work [43], [44], [45]. Examining structural elements of sexual risk still appreciates variations in individual risk behaviors and decisions, but also situates individual behaviors in appropriate larger social contexts to interpret sexual risk. Based on input from local STI physicians/managers [46] and low-income sex workers, we used a behavioral-structural framework [42]. This framework examines the larger social and structural factors that influence individual sexual behaviors without making assumptions about sex worker agency. In the context of female commercial sex in China, key social structures for women include sex venue relationships with managers (if present), outreach worker relationships, and fellow sex worker relationships. This choice of framework guided our decision to include both female sex workers and health outreach workers as part of the study.

A total of 34 low-income sex workers were interviewed in three Pearl River Delta (Guangdong Province) municipalities during two phases from December 2009 through July 2010. The study population was women who sold sex in the past month for less than five US dollars per client, referred to as low-income sex workers. An expanding literature supports increased sexual risk among low-income female sex workers in China [47]. This purposive sample was selected to include women who worked in a number of different sex venues, had various relationships with pimps/managers (managed by gatekeeper or working independently), and had various levels of social support. These particular purposive sampling dimensions were selected based on our behavioral-structural framework and prior research in China suggesting that sex venue [4], [40], occupational characteristics [48], and social support [4], [39] are important determinants of sexual risk. Participants were recruited by the local sex worker advocacy organization identifying women during syphilis/HIV outreach campaigns, and this group was supplemented by women referring their friends who also sold sex.

Each semi-structured interview was conducted by a trained interviewer and a note-taker/translator. The interviewers were Chinese or Chinese-Americans who received training in interview techniques. One of the three interviewers was involved in local health outreach campaigns and already known to the participants while two other interviewers were not known to participants before the study. After obtaining oral consent from the participants, the interviewer administered a semi-structured survey that contained the following content domains: social and demographic characteristics, social networks (network characteristics, material aid, physical aid, sense of belonging, health-specific guidance), condom norms (condom use, condom negotiation), health seeking-behaviors (STI/HIV testing, clinic preferences), and exposure to violence. The choice of domains was informed by our behavioral-structural framework. The interview guide was developed in collaboration with the Guangdong Provincial STD Control Center and a formally organized sex worker NGO in a nearby region. Names and contact information were not collected and each interview was assigned a unique numerical identifier. Participants were given a small gift and provided sexual health and violence prevention resources. Interviews lasted 45–60 minutes and three participants had a second follow-up interview. The purpose of the second interview was to expand on topics that were not sufficiently developed during the first interview.

A total of 28 outreach members were interviewed, with 13 individuals having both outreach and physician responsibilities currently or in the past. Outreach members were local public health officials tasked to sex worker outreach programs who had substantive experience conducting outreach programs specifically designed to improve sex worker health. Interviews occurred following the female sex worker interviews so that themes from the female sex workers could be discussed among outreach members. The substantive format of the outreach member interviews was analogous to female sex worker interviews.

If participants were willing to have their interviews recorded, data was digitally recorded and transcribed into Mandarin Chinese. If women were not willing to be recorded, a research assistant took notes during the interview. All textual analysis was done in Mandarin Chinese. The analysis started with KW and JT independently reviewing the transcripts to develop a list of main themes related to the role of social networks in women's sexual risk taking. Data were collected in two waves to inform recruitment and purposive sampling and further distinguish emergent themes. Themes were identified based on the data. Coding of data was undertaken in two phases – first to identify major broad themes and then to delineate subtopics of importance within each of the broad themes. Coding was done without software and descriptively organized by theme.

## Results

Among the 34 low-income sex worker participants, the average age was 38 years old. Women were from Yiyang City in Hunan Province (13), Taojiang County in Hunan Province (10), Jiangxi Province (3), Sichuan Province (3), Guangdong Province (2), Chongqing Municipality (1), and Guizhou Province (1). All women were married and had at least one child.

### Laoxiang social networks

The terms *laoxiang* or *laoxiang jiemei* were translated as hometown social network contact, referring to the women who migrated from the same region. All the women reported having *laoxiang* sisters in the area, with a range of 3–15, median of nine. This *laoxiang* social network included both women who sold sex and those who did not, but all of these network members lived in the same urban municipality as the participant. Many women also reported that they would either lend (cases 28, 29, 35) or borrow (cases 28, 29, 33, 35) money from *laoxiang*. One woman stated, “We all band together and chip in for those [*laoxiang*] who need money” (case 28). The importance of these hometown social ties was also observed by outreach members who noted, “*Xiaojie* (sex workers) will believe and trust each other if they come from the same place” (O18). *Laoxiang* networks represented a critically important theme that organized women's lives and influenced health behaviors, including obtaining and using condoms, managing clients who refuse condom use, promoting health-seeking behaviors and STI/HIV testing, and mitigating the effects of anti-prostitution campaigns. Women frequently introduced their *laoxiang* sisters either generally to selling sex or more specifically to the local sex venues and managers/pimps (cases 13, 14, 16, 22, 23, 24, 25). These social relationships included women who knew their *laoxiang* prior to migration and those who met *laoxiang* only after migrating to the urban destination.

### Laoxiang influence on condom use

There were several mechanisms whereby *laoxiang* sisters influenced condom use: promoting wholesale condom purchasing; mediating condom use with clients; and providing options for managing clients who refused condom use. The collective organization of *laoxiang* sisters facilitated wholesale condom purchasing (Supporting Information S1). Sex workers reported receiving condoms from outreach members during specific programs, but there were no long-term programs or STI clinic based distribution programs. Several outreach members reported that condom distribution programs were sporadic and linked to provincial programs with specific end dates (OM 4, OM 18). In order to ensure a consistent supply of condoms, a group of *laoxiang* sisters collectively negotiated less expensive condom prices through a wholesale operation (case 26, 27, 28; Supporting Information S1). Women reported that purchasing at least a certain amount allowed them to save money and had the added benefit of free delivery to their venue (case 27). Although it was not clear from this sample if non-*laoxiang* contacts participated in the wholesale purchasing, the social network allowed women to more efficiently purchase condoms.

Female sex workers who shared a common hometown consulted each other about condom use and condom negotiation. Several women reported that they first learned about condom use from another woman with the same hometown (cases 26–29, 31–32, 35). One woman noted, “When I first came here to start working, it was [*laoxiang* sister name] who taught me how to use condoms. She said that this was the hygienic way” (case 27). However, the same *laoxiang* networks did not always promote condom use: “Sometimes *laoxiang* talked about advising against condom use. A client purchasing [sex] who is unwilling to use a condom ….can be a fine source of money as well” (case 27). At the same time, two women reported only positive influences on condom use from their *laoxiang* (cases 29, 32).

Male clients who absolutely refused condom use would encounter conflict in such tightly knit communities, resulting in arguments, collective responses, and sometimes police involvement. Women reported that *laoxiang* structures were important in backing them up when facing belligerent clients (cases 12, 20, 24, 27–33; Supporting Information S1). One woman reported, “*Laoxiang* sisters talk about difficult clients and then next time we see them [difficult clients] we take extra care…We help each other cope with this kind of client. We have so many *laoxiang* sisters, there is no need to worry” (case 27). If a client tried to force non-condom use, one woman reported that she would shout to her nearby *laoxiang* (case 12) while another would use her mobile phone to relay the message to her social network (case 24). This analysis was not able to differentiate the balance between giving and providing help with difficult clients, although four women identified an older *laoxiang* (cases 20, 24, 27–28) female sex worker who was particularly savvy with providing help to women in condom negotiation.

This level of commitment to one's *laoxiang* social network did not extend to other women selling sex at the venue unless they were also from the same hometown (cases 27, 30, 32, 33). When asked about helping a non-*laoxiang* sex worker from the same venue with a violent client, one woman responded, “I would call the police, I can't get too involved in other people's business” (case 32).

### Laoxiang influence on capacity to deal with anti-prostitution campaigns

*Laoxiang* social networks served as a social support system during anti-prostitution campaigns. Women from *laoxiang* social networks would call each other if police were soon to implement a Strike Hard campaign (cases 1, 7). This advance notice of police campaigns included the following three steps: social network members maintained relationships with both police and the local neighborhood committee in order to gain access to information, hometown social networks communicated with each other via mobile phones preceding campaigns, and *laoxiang* social networks were strengthened during police campaigns since women would often engage in communal activities (card playing, mahjong, etc.). One sex worker explained how the system worked: “A few hometown sisters are policewomen and they reveal the timing [of Strike Hard Campaigns], telling hometown social networks to take notice” (case 2).

Two members of a *laoxiang* network reported that their connections with the local neighborhood committee were instrumental in anticipating anti-prostitution campaigns (cases 12, 15). These observations were further supported by four outreach workers who noted that women would call their *laoxiang* prior to anti-prostitution raids. One woman explained the close local relationships: “Before [police] investigated here to check documents [residency permits], but during the last 2–3 years none of the police have come by. Now the local neighborhood committee investigates and the police only care about finding clients near the lake. Receiving clients here is not tightly controlled [by the police], they also know that we provide services to some senior officials” (case 16). Another woman from the other hometown network further explained: “Last year the police came to investigate, 5–6 police officers, looking up and down my apartment, checking my documents, and then leaving. But now before the [anti-prostitution] campaigns I get a phone call” (case 3). The *laoxiang* network created a sense of safety: “I don't fear the police, [*laoxiang*] are all around, this area is all *laoxiang* sisters (case 8).

Not all *laoxiang* networks had forewarning of police Strike Hard anti-prostitution campaigns. One woman noted: “I don't know [when police campaigns will happen], they are all irregular. I rely on luck and running to quieter areas” (case 21). One sex worker explained: “I don't [trust anyone], I think society now is all false, and no one is worth trusting” (case 25). Participant observation noted that women at a larger sex venue with smaller social networks had less forewarning of anti-prostitution campaigns and less trust in those working at the same venue. This site also had been subject to more regular anti-prostitution campaigns resulting in detention and fines.

### Laoxiang influence on health seeking behaviors

*Laoxiang* social networks also had important implications for health seeking behaviors and developing trust in local sexual health services. Sex worker health programs were organized almost entirely by local government-run public health programs (OM 8). There was limited sex worker NGO development despite large numbers of sex workers in each of the urban regions. One outreach member described, “We have a Red Cross NGO that helps with *xiao jie* [female sex worker] outreach, but there are not special *xiaojie* NGOs that only work with *xiaojies*” (OM 18). The challenges faced by trying to organize an NGO were described: “Finding people to do the [NGO] work was hard. The energy had to be within you. We had to find people who were able to talk with sex workers, who understood them. Rather persistent people. You couldn't just do it one or two days and stop. It was very difficult” (OM 8).

Sex worker outreach programs were complicated by their uncertain relationship to the police: “The difficulties were great, especially when we started. The local police dispatched people to drive us out. They didn't understand. Sex workers would take our condoms and throw them on the ground…*Sao huang* (anti-prostitution raids) is a big problem” (OM 26).

Sex workers looked to their *laoxiang* social network contacts for advice on getting STI/HIV tests (cases 5, 9 Supporting Information S2), seeing a specific physician (case 4), attending a specific clinic (cases 1, 3, 5, 6, 15), or self-treatment (cases 1, 2). Several women reported that they would accompany (cases 28, 29, 30, 32, 35) or be accompanied (cases 27, 31) by *laoxiang* when seeking health services. One participant reported that she trusted a local gynecologist who was from the same city in Hunan Province (case 1) and another sought medical advice from an old hometown network friend who is a physician (case 3). The *laoxiang* social networks helped to alleviate distrust of the medical system, opening up opportunities for STI/HIV testing (Supporting Information S2). One woman remarked, “The first time I saw a doctor was with my *laoxiang* sister. Because I had just arrived and didn't know the place well, she accompanied me to a friendly clinic….Now I know all the places [clinics]. I often take *laoxiang* sisters to see the doctor. Before they wouldn't dare to get treated but now I accompany them” (case 31). The theme of *laoxiang* sisters introducing local clinics emerged from a number of women (case 31, 33, 34).

The connection between *laoxiang* social network members also influenced willingness to be tested for STI/HIV once at the clinic: “I most trust [case 9], her and the other *laoxiang* social network members who sell sex, we often go out for fun, we know each other well. Each time I get tested it is because of [case 9]” (case 11). One of the advocacy workers framed the promotion of syphilis/HIV testing in the context of *laoxiang* social networks, telling sex workers that many of their Hunanese sisters had already been tested (OM 4). Hometown social networks were a valued source of information about medications, clinics, and STI/HIV testing preferences.

## Discussion

Female sex workers have been the subject of many network studies describing their influence on disease transmission [49], [50], but few studies have analyzed the positive influence of female sex worker networks on sexual health [43]. Our data suggest that *laoxiang* ties may establish healthy behavioral norms, create relationships with outreach group members and physicians, and help to create a local environment conducive to sexual health. While other studies have investigated social connections between sex workers in China [37], [38], they have not focused on social networks. To the best of our knowledge, this is the first published study to examine sex worker social networks that could be used to promote sexual health in China. Especially in the context of expanding biomedical options for HIV prevention, ranging from female condoms to pre-exposure prophylaxis, a nuanced understanding of female sex worker social networks may be crucial for translating innovative strategies into effective widespread public health campaigns. Government, non-governmental, and advocacy groups charged with organizing sex worker HIV prevention campaigns could benefit from understanding the social networks of female sex workers.

Our finding that Chinese social connections influence condom use and sexual risk is consistent with research from a number of settings within and outside China. Two studies of Chinese female sex workers found that venue-level peer influences were important predictors of individual condom use [38], [51]. Other Chinese female sex worker studies have identified venue-level factors as important determinants of HIV infection [52] and STI infection [53], but have not formally analyzed underlying network structures. A Canadian study showed that social network norms influence individual condom use [54]. A Brazilian analysis of female sex workers found that increased participation in social networks was associated with a decrease in frequency of unprotected sex [55].

The function of *laoxiang* social networks in promoting health seeking behaviors, encouraging STI/HIV testing, and linking women to outreach workers has not been described before in China. The literature on how to encourage sex workers to trust local outreach staff and regularly attend clinics is sparse. An STI clinic-based study in China found that individuals who were accompanied were significantly more likely to accept HIV testing [56]. Studies outside China have also demonstrated social network influences on HIV testing [57], [58].

While *laoxiang* networks seemed to have a net positive influence on condom use and STI/HIV testing, we found several instances where social networks were used to promote risky behaviors or avoid STI/HIV testing. Both community popular opinion leader and peer-based behavioral interventions assume that influential sex workers have less risky behaviors, but our data provide empirical examples to challenge this assumption. Much research outside of the sexual health field has described the spread of unhealthy behaviors through social networks. Unhealthy behaviors ranging from smoking, over-eating, drug use, and binge drinking may be normalized in the context of social network relationships and norm establishment [5], [59], [60], [61]. More research on the influence of social networks in creating and changing sexual behavioral norms is critical.

This research has several limitations. First, our study assumed that *laoxiang* social connections are stable over time, but women's identification with their home region may be more dynamic. For example, a woman from one region may choose to identify with her province, prefecture, city, or district depending on individual relationships and strength of network ties. If a new group of female sex workers came from a different district within the same city, the home affiliation could widen to incorporate the new group or remain unchanged. Second, the emergent civil society in China does not preclude formal nongovernmental organizations from developing, it simply slows the development of sex worker advocacy organizations. The social connections that were observed may be useful in the development of sex worker organizations among some groups of women. Third, although we found an association between strong *laoxiang* social networks and curbed effects of anti-prostitution campaigns, this study did not analyze police/sex worker connections over time and cannot make claims about causation. Finally, this study only analyzed qualitative data from a small group of female sex workers and outreach members in one region, so generalizations to other settings should be made with caution.

Social networks may be a useful organizational unit to design, tailor, and implement STI/HIV prevention programs for sex workers [62]. One qualitative study from Brazil also found that organizing sex worker programs around collective identities rather than sex worker identities may be a stronger force for health promotion [63]. Especially in regions without a strong civil society, targeting STI/HIV prevention to cohesive social groups may be able to more effectively and sustainably reach marginalized groups. The point should also be made that leveraging these social ties for STI/HIV prevention does not circumvent civil society or mean a lesser role for NGOs in sex worker health programs. On the contrary, designing sex worker health programs based on social ties may provide an organizational foundation to develop new nongovernmental organizations and improve advocacy and outreach. Further research is needed on the social networks of female sex workers in order to build a stronger foundation for biomedical and behavioral interventions.

## Supporting Information

Supporting Information S1 ***Laoxiang*** **female sex worker influence on condom use and violence mitigation.**
(DOC)

Supporting Information S2 **Promoting linkage to services and STI/HIV testing.**
(DOC)
