# Supplementary material for: Female Sex Worker Social Networks and STI/HIV Prevention in South China
Source: PLoS One. 2011 Sep 13;6(9):e24816. doi: 10.1371/journal.pone.0024816 (PMC3172283; doi:10.1371/journal.pone.0024816)
Supplement: Supporting Information S1 — Laoxiang female sex worker influence on condom use and violence mitigation. (DOC) [file pone.0024816.s001.doc]

| **Supporting Information S1 *Laoxiang* female sex worker influence on condom use and violence mitigation** |
| --- |
| **Extract 1 – Negotiating wholesale condom purchase**  We [*laoxiang*] always chat about where is the best place to buy condoms, *laoxiang* sisters introduce the system, they call me to try it, and after I try I can find this person [wholesaler]. After I use all my condoms I give them a call and don’t have to go out to buy condoms (case 28)  **Extract 2 – Mediating condom use**  Sometimes *laoxiang* sisters together discuss [condom use], and if there is an unwilling client just find another client. They told me not to accept clients unwilling to use condoms, so I don’t dare. I will only accept a client if they are willing to use a condom (case 28)  An older woman from my hometown advised me to use condoms….She said that this [selling sex] is not safe, not using condoms makes it easier to get disease and I haven’t gotten any diseases in my whole life (case 32)  **Extract 3 – Managing clients who refuse condom use**  In the small chance that [clients] act crude, we don’t do business and go outside. When the situation doesn’t feel right, we quietly phone to notify *laoxiang* sisters. We all do it this way, and mutually keep an eye on one another (case 24)  He [client] did not want to use a condom, I wasn’t willing to do this and told him to leave, and he was terrible to me. He said, “You had better be careful and watch yourself.” But I wasn’t afraid since here we have so many *laoxiang* sisters. If he dared to do this, he wouldn’t be able to leave. We here look out for each other – if anyone has something come up, just give a holler and everyone would come to help (case 12)  **Extract 4 – Prevention and mitigation of violence**  Yesterday a client was drunk, steaming off that [he would] not give money, fighting with me. In the end, the *laoxiang* sisters emerged to take a stand and cursed him, saying, “We here, if you don’t pay up, you can’t leave…” (case 20)  To protect yourself, pick clients by their looks and expression in their eyes, not accepting the fierce ones. Sometimes *laoxiang* sisters will call attention to a client who should be avoided (case 6) |
