# Supplementary material for: Female Sex Worker Social Networks and STI/HIV Prevention in South China
Source: PLoS One. 2011 Sep 13;6(9):e24816. doi: 10.1371/journal.pone.0024816 (PMC3172283; doi:10.1371/journal.pone.0024816)
Supplement: Supporting Information S2 — Promoting linkage to services and STI/HIV testing. (DOC) [file pone.0024816.s002.doc]

| **Supporting Information S2 Promoting linkage to services and STI/HIV testing** |
| --- |
| **Extract 1 – Linking *laoxiang* sisters to outreach teams**  Just today I was doing an outreach program and was trying to talk to a *xiaojie*, but I could tell from the look on her face that she was not listening. Then three of her Hunan hometown sisters came by and took the condoms, but didn’t say anything. After that the woman accepted the condoms and was much more open. If you trust your *jiemei*, then you can trust our outreach team (OM 18)  Yesterday at an entertainment center the *xiaojie* manager was not interested in our outreach team, so all of the *xiaojie* at that site refused to accept condoms or pamphlets. I went and spoke with some of the *xiaojie* and it took several outreach trips to get them to listen…. Finally several of the *xiaojie* there agreed to be tested for syphilis [on-site], so the remaining *laoxiang* were more willing to be tested (OM 17)  **Extract 2 – Encouraging STI/HIV testing**  *Female sex worker perspective:*  At first I wouldn’t dare get blood drawn [for an HIV or syphilis] test… but then several *laoxiang* were tested. They [*laoxiang*] said the hospital [to get tested] was nearby, wouldn’t cheat patients, and was furthermore free. I went straight away to get tested (case 5)  Many of our *laoxiang* were helped by the advocacy group to get [syphilis] tested, and after getting tested there was nothing to fear, if tests were positive for syphilis then just get treated, and if tests were negative then you could relax. So [*laoxiang* name] accompanied me to get tested (case 9)  If I don’t feel well and need to see a doctor, I will find them [*laoxiang*]. They are all willing to accompany me to see a doctor. Sometimes they have problems and need my help, and sometimes I also need their help. We are from a single place and mutually help each other (case 12)  *Outreach member perspective:*  Small groups are more willing to accept [rapid on-site] syphilis testing. Power in numbers, but this can also backfire. If one *xiaojie* obviously refuses to cooperate with the outreach team, then often all of the *xiaojie* will refuse to participate. *Laoxiang* can have a strong effect on whether or not a woman ends up accepting a syphilis test or not accepting a syphilis test. If one woman accepts testing, her hometown sisters are more willing to get tested. If one woman refuses testing, her hometown sisters are less willing to be tested. The other week I met a woman that had refused before when she was with her friends but then I saw her with a different group she was willing to be tested (OM 18)  If a hometown sister has been tested, then it is much easier to get her hometown sisters to be tested. The situation of not having *laoxiang* help can be challenging. This can be extremely difficult to do outreach with *xiaojie* who do not have *laoxiang*. *Laoxiang* sisters trust their own hometown sisters. After all, they are their own. Very few sex workers come all alone, most come to the city with their hometown sisters (OM 17) |
